# Effects of Low Doses of Ionizing Radiation Exposures on Stress-Responsive Gene Expression in Human Embryonic Stem Cells

**DOI:** 10.3390/ijms15010588

**Published:** 2014-01-06

**Authors:** Mykyta Sokolov, Ronald Neumann

**Affiliations:** Nuclear Medicine Division, Department of Radiology and Imaging Sciences, Clinical Center, National Institutes of Health, 9000 Rockville Pike, Bethesda, MD 20892, USA; E-Mail: rneumann@mail.nih.gov

**Keywords:** human embryonic stem cells, ionizing radiation, low dose exposures, gene expression changes

## Abstract

There is a great deal of uncertainty on how low (≤0.1 Gy) doses of ionizing radiation (IR) affect human cells, partly due to a lack of suitable experimental model systems for such studies. The uncertainties arising from low-dose IR human data undermine practical societal needs to predict health risks emerging from diagnostic medical tests’ radiation, natural background radiation, and environmental radiological accidents. To eliminate a variability associated with remarkable differences in radioresponses of hundreds of differentiated cell types, we established a novel, human embryonic stem cell (hESC)-based model to examine the radiobiological effects in human cells. Our aim is to comprehensively elucidate the gene expression changes in a panel of various hESC lines following low IR doses of 0.01; 0.05; 0.1 Gy; and, as a reference, relatively high dose of 1 Gy of IR. Here, we examined the dynamics of transcriptional changes of well-established IR-responsive set of genes, including *CDKN1A*, *GADD45A*, *etc.* at 2 and 16 h post-IR, representing “early” and “late” radioresponses of hESCs. Our findings suggest the temporal- and hESC line-dependence of stress gene radioresponses with no statistically significant evidence for a linear dose-response relationship within the lowest doses of IR exposures.

## 1. Introduction

The biological effects of low doses (LD) of IR, especially as related to any health effects and risk assessment in humans, remain in the spotlight of intense scientific research, generating a substantial level of controversy (for reviews, see [1,2]). The importance of such studies stems from the fact that human exposures to LD IR emanating both from background radiation sources (Earth’ crust radioisotopes, cosmic rays, *etc.*), and various types of human activities (nuclear power industry, radioactive waste, radiologic accidents, diagnostic testing IR exposures in clinical practice, *etc.*) are essentially inevitable. Low dose exposures are without doubt far more common than exposures to high doses (HD) of IR (mostly from therapeutic treatments) [3]. In general, one of the key problems in radiation effects research is how to extrapolate the well-established data on normal tissue damage and cancer risk assessment from HD IR exposures to LD range (equal to or less than 0.1 Gy). Epidemiological data imply that IR exposures of more than 0.2 Gy increase the risks for cancer and other pathologies [4]. In contrast, a remarkable lack of consensus in the scientific literature regarding the biological effects of LD still persists. For example, the latest “Biological Effects of Ionizing Radiation” (BEIR VII) report stated that the totality of available biological data remains consistent with a “linear, no-threshold” (LNT) hypothesis [5]. The essence of the LNT hypothesis postulates that even the smallest doses of IR could increase the risk for carcinogenesis. On the other hand, the recent French Academy of Sciences report tends to emphasize ever-mounting evidence for non-linearity in biological effects of LD IR effectively questioning the LNT hypothesis [6]. The occurrence of non-targeted effects of IR, often observed following LD IR, such as adaptive responses, bystander effects and low-dose hypersensitivity, and the potentially beneficial hormetic effects of at least some LD exposures, presents additional levels of complexity to LD radiobiology [7–11]. A clarification of the shape of the curve for dose-response effects within the LD range would lead to more appropriate estimates of LD risks; and, as a result, would firmly establish public policy standards regarding radiologic medical examinations, radioprotective measures, *etc.*, potentially saving valuable financial resources.

Past research in radiobiology has convincingly established that one of the integral parts of the biological responses to IR exposures is global changes in gene expression, especially those associated with genotoxic stress responses [12–14]. Previous studies were focused upon both HD and LD IR transcriptional responses *in vitro* [15–19], *ex vivo* [20,21], and *in vivo* settings in humans [22]. What emerges from these and other reports is that LD IR responses, including gene expression alterations, are highly genotype, cell type, and tissue-dependent, with a remarkable degree of variability both between individuals and different cell types [23–28]. Some estimates suggest that the human body contains approximately three hundred various types of differentiated cells. The transcriptional radioresponses for at least some of them are astonishingly distinct. For example, one of the best known IR-responsive genes, namely *CDKN1A*, was found to be robustly upregulated in fibroblasts across the wide range of IR doses, but not in keratinocytes [26]. To address the problem of differential IR-induced gene expression changes among various normal human cells, we developed a novel human embryonic stem cell (hESC)-based culture model to examine radiobiological effects in human cells. It is only recently that such studies into transcriptional changes in IR-exposed hESCs began to address hESC radioresponses [29–32]. However, the doses used in these reported studies were in the range 0.4–4 Gy. Currently there is a lack of knowledge of how hESCs respond to LD of IR.

In the present study, our goal was to fully examine gene expression alterations in a panel of several hESC lines following exposures to LD IR doses of 0.01; 0.05; and 0.1 Gy. As a positive control for reference, we also used the relatively high dose of 1 Gy of IR. We aimed to elucidate the dynamics of transcriptional changes of a well-established IR-responsive set of genes at 2 and 16 h post-IR, representing “early” and “late” hESC radioresponses, respectively. Our findings suggest both a temporal- and a hESC line-dependence of stress gene radioresponses with no solid evidence for a linear dose-response relationship within the range of LD IR by these hESCs.

## 2. Results and Discussion

It has long been a goal in radiation biology, radiation oncology, and radiation protection to identify biomarkers of IR exposures that can comprehensively predict the ultimate radioresponses of normal and tumor tissues, as well as to allow the triage of people following radiation incidents of various kinds (nuclear power plant accidents, potential “dirty bomb” terrorist attacks, *etc.*). Recently, powerful high-throughput genome-wide profiling approaches significantly expanded the repertoire of technical tools that radiation researchers can employ to discover such biomarkers. A body of scientific evidence points to a relatively limited number of genes consistently showing IR-responsiveness across different human genomes under different exposure scenarios, among them *CDKN1A* [14,22,33–40], *GADD45A* [41–44], *BTG2* [36,37,45,46], *BBC3* [38,47,48], *PCNA* [17,46,49], *SESN1* [26,47], *IER5* [44,50,51], *GDF15* [38,52,53], and *PLK3* [38,54,55] representing the most studied. Moreover, all or some of these genes were shown to constitute an essential part of a consensus IR dose-response signatures reported previously [39,47,56]; and subgroup of these genes, namely *CDKN1A*, *GADD45A*, *PCNA*, and *BBC3* discriminated profiles of IR-responsive biomarkers from those triggered by other stimuli, such as an inflammation [57].

Many of the gene expression studies that examined IR-responsive sets of genes, and characterized their profiles after IR exposures concluded that there is a high degree of variability in radioresponses across various individual normal tissues and different types of differentiated cells [26,28]. Among the genes we examined in our present study, *CDKN1A* was robustly induced in fibroblasts within the wide range of IR doses (0.1–10 Gy), but not in keratinocytes. In marked contrast, expression of *SESN1* remained at basal levels up to 1 Gy of IR exposures in fibroblasts, but was elicited even by LD IR of 0.1 Gy (more than 3-fold up) in keratinocytes [26]. Importantly, normal tissues from some donors may display only a minimal radioresponse [26]. Also, we previously found a very limited overlap in gene expression changes between human keratinocytes and fibroblasts after DNA-incorporated isotope IR exposures [14]. Only a few alterations were found in common between dermis and epidermis in 3-D human tissues after IR [28]. Therefore, it is imperative to use a relevant human model system in which to study radioresponses with minimal interference from the abovementioned confounding factors. We believe hESC cultures may provide such a useful model system. However, it is only recently that attempts began to comprehensively characterize the radioresponse of hESCs [29–32,58–60].

**Figure 1.**
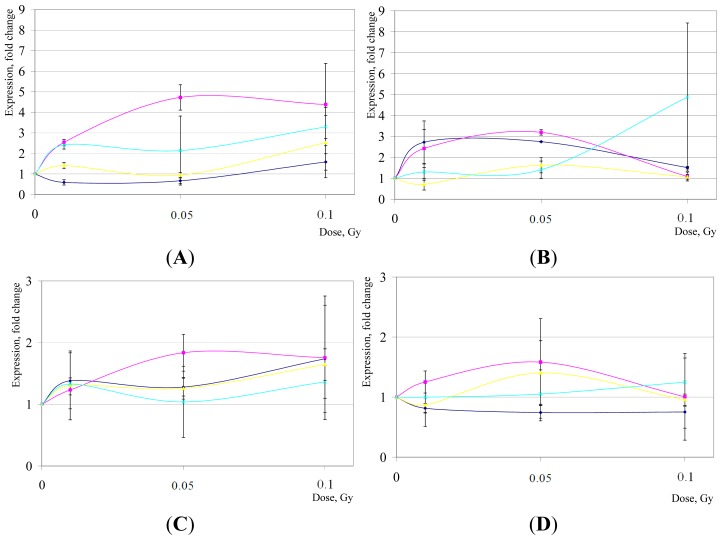
*CDKN1A* (**A**,**B**) and *GADD45A* (**C**,**D**) gene expression changes in hESCs after LD of IR exposures. Shown are mean relative quantities (±SD) for cell cultures post treatments compared to sham-IR. In violet, H1 hESC line; in pink, H7; in yellow, H9; and in blue, H14. (**A**,**C**), gene expression changes 2 h post-IR; (**B**,**D**), 16 h post-IR, respectively.

Published data regarding the expression of *CDKN1A* in IR-exposed hESCs are largely inconsistent. For example, the same group reported that *CDKN1A* was overexpressed either about 250-fold [61] or only 15-fold [58] in H1 hESCs after 5 Gy of IR (2 h post-exposures) compared to controls. Other reports showed that *CDKN1A* induction is observed only after HD IR (2–4 Gy, about 2–2.3-fold relative to control); and the modest doses of IR as low as 0.4 Gy fail to trigger any overexpression of this gene in H9 hESCs [30]. However, our previous data implied that a dose of 1 Gy of IR is sufficient to elicit a robust upregulation of *CDKN1A* in H9 hESCs (about 5.8-fold at 2 h post-IR, and 1.9-fold at 16 h) [31]. Interestingly, UV radiation exposures were shown to result either in a decrease in expression of transcripts of *CDKN1A* in undifferentiated H1 hESCs [62], or, in a marked contrast, a robust *CDKN1A* increase (about 27-fold upregulation in low-passage hESC cultures) [63]. Therefore, additional studies to examine *CDKN1A* gene expression alterations in hESCs are highly warranted, in part at least, to clarify the apparent discrepancies found in different reports in the literature. Our present data suggest that *CDKN1A* expression fluctuates within the LD range (Figure 1). In general, the pattern of expression changes for this gene appeared to be complex. However, except for the H14 hESC line at 2 h post-LD IR (*p* < 0.05), none of these alterations has proven to be statistically significant compared to sham-IR (*p* > 0.05).

*GADD45A* responds to environmental stresses by activating the p38/JNK pathway. *GADD45* gene is known to demonstrate a complex response to IR exposures; some TP53 wild-type cells do not show any overexpression of *GADD45* after IR [22,64]. The existing data on *GADD45* expression in hESC are scarce but it was reported that doses as low as 0.1 Gy are capable of eliciting an overexpression of this gene [30]. There seemed to be a dose-dependence within 0.1–1 Gy of IR exposures, which is above the range of LD IR [30]. Here, we observed some modulations in expression of *GADD45A* in hESC within 0.01–0.1 Gy dose range, but with no clear indication for a linear dose-response relationship (Figure 1). Moreover, except for H1 hESC at 16 h post-LD IR (*p* < 0.05), the changes were statistically insignificant given a large variability in expression levels between different populations of hESC cultures (*p* > 0.05).

**Figure 2.**
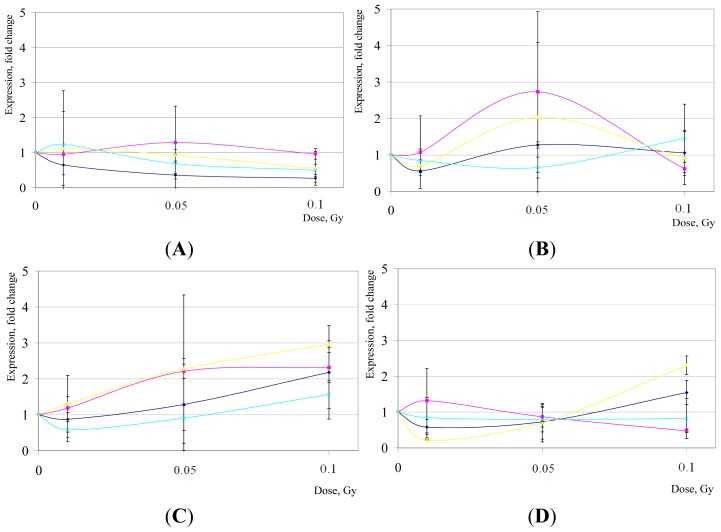
*IER5* (**A**,**B**) and *SESN1* (**C**,**D**) gene expression changes in hESCs after LD of IR exposures. Shown are mean relative quantities (±SD) for cell cultures post treatments compared to sham-IR. In violet, H1 hESC line; in pink, H7; in yellow, H9; and in blue, H14. (**A**,**C**), gene expression changes 2 h post-IR; (**B**,**D**), 16 h post-IR, respectively.

IR-induced modulations of *IER5* expression were implicated as affecting the radiosensitivity of non-stem human cells through perturbations of cell-cycle checkpoints, especially of G2/M checkpoint [50], by inhibiting cell proliferation. Importantly, G2/M checkpoint is fully operational in hESCs, and is robustly induced by HD IR exposures in hESC cultures [65]. Our data suggest that LD IR exposures fail to trigger robust changes in *IER5* expression in the four hESC lines that we analyzed; with the exception of an early response (2 h) in the H1 line (*p* < 0.05), alterations in *IER5* expression were statistically insignificant (*p* > 0.05) (Figure 2).

*SESN1* is a known TP53 target gene regulating cell growth and proliferation; however, *SESN1* response to LD IR in hESC awaits characterization. Only a late response in the H14 line showed significant changes compared to controls (*p* < 0.05); for all other hESC lines and conditions, we found a lack of significant induction for this gene post-LD IR (*p* > 0.05) (Figure 2).

**Figure 3.**
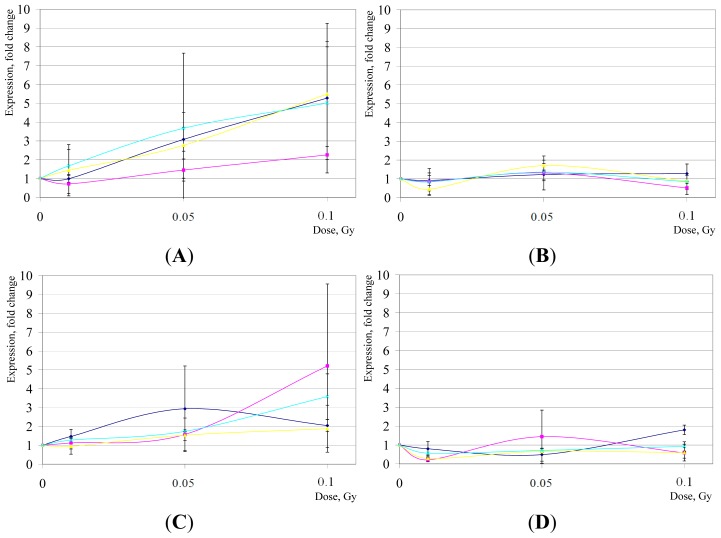
*GDF15* (**A**,**B**) and *PLK3* (**C**,**D**) gene expression changes in hESCs after LD of IR exposures. Shown are mean relative quantities (±SD) for cell cultures post treatments compared to sham-IR. In violet, H1 hESC line; in pink, H7; in yellow, H9; and in blue, H14. (**A**,**C**), gene expression changes 2 h post-IR; (**B**,**D**), 16 h post-IR, respectively.

*PLK3* has been demonstrated to be an important mediator of the cellular responses to genotoxic stresses [66], at least in part through the TP53-p21 pathway [67] and the NF-κB pathway [68]. Overexpression of *PLK3* can result in cell cycle arrest and apoptosis, affecting G1/S transition [69], perturbing microtubule integrity, and eliciting G2/M arrest [70]. *GDF15* is a member of the TGF-β superfamily responding to various stresses [71]. Our analysis indicates that some fluctuations in expression levels observed for both *PLK3* and *GDF15* within LD range of IR at 2 h seemed to be rather transient since no robust changes were found at 16 hr post-IR (*p* > 0.05) (Figure 3).

**Figure 4.**
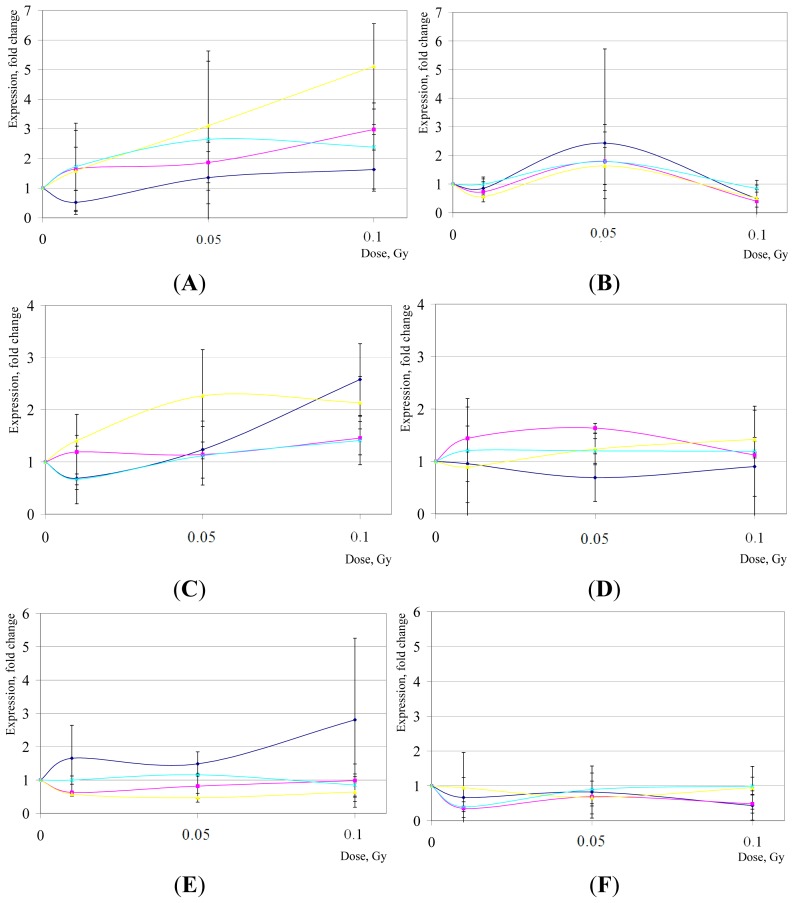
*BTG2* (**A**,**B**), *BBC3* (**C**) and (**D**), and *PCNA* (**E**) and (**F**) gene expression changes in hESCs after LD of IR exposures. Shown are mean relative quantities (±SD) for cell cultures post treatments compared to sham-IR. In violet, H1 hESC line; in pink, H7; in yellow, H9; and in blue, H14. (**A**,**C**,**E**), gene expression changes 2 h post-IR; (**B**,**D**,**F**), 16 h post-IR, respectively.

*BTG2* is a known tumor suppressor preventing cell proliferation; it is transiently induced by oxidative stress through both TP53 and NF-κB pathways [72], stimulating repair of DNA double-strand breaks (DSBs) [73] and cellular antioxidant defenses [74]. Our results indicate that the modulations in *BTG2* expression following LD of IR exposures are highly temporal- and cell line-dependent in hESCs (Figure 4). At 2 h post-LD IR in H14 hESC line, we observed significant changes in expression of *BTG2* compared to sham-IR exposures (*p* < 0.05); however, in other hESC lines *BTG2* expression alterations were found to be not statistically significant (*p* > 0.05).

One of the best-known effectors of apoptosis is *BBC3* which belongs to the BH3-only pro-apoptotic family, cooperating with effectors to induce mitochondrial permeabilization and, eventually, programmed cell death. *BBC3* is induced by DNA damage [75], and is a target of both the TP53 [76] and NF-κB [77] transcription factors. After LD of IR exposures, there were no statistically significant changes in expression of *BBC3* compared to sham-treatment (*p* > 0.05), except for at 16 hr post-IR in H14 hESC line (*p* < 0.05) (Figure 4).

Independent groups have shown that the majority of hESCs are at S phase at any given time [61,65]; therefore, it seems important to examine the changes in expression of *PCNA*, a cofactor of DNA polymerase delta, after LD IR exposures of hESC cultures. Here we demonstrate that no robust changes in *PCNA* transcript levels could be found in hESCs as part of an early response to IR (*p* > 0.05), except for H9 hESC line (*p* < 0.05); at 16 h post-exposures, we observed a modest downregulation of *PCNA* in H7 hESC line (Figure 4).

**Figure 5.**
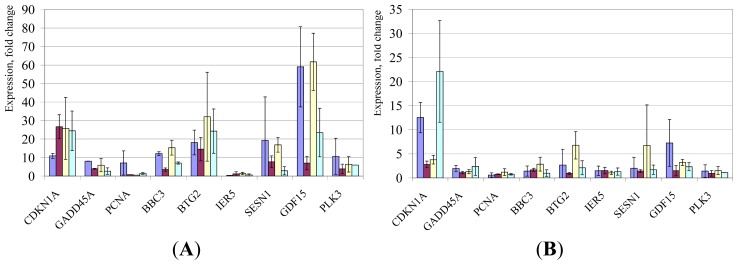
Stress-responsive gene expression changes in hESCs after HD of IR exposures (1 Gy). Shown are mean relative quantities (±SD) for cell cultures post treatments compared to sham-IR. In blue, H1 hESC line; in maroon, H7; in yellow, H9; and in green, H14. (**A**), gene expression changes 2 h post-IR; (**B**), 16 h post-IR, respectively.

To compare the effects of LD IR and HD IR exposures, we performed an analysis of changes in stress-responsive gene expression in hESC after 1 Gy of IR (Figure 5). The majority of genes showed a robust upregulation, especially *CDKN1A*, *GDF15*, *SESN1* and *BTG2*. The magnitude of alterations in *CDKN1A* after HD IR was comparable to those observed for irradiated hESC before [58,63], and vastly exceeds that following LD IR. We recently published that *GADD45A* is robustly upregulated in H9 hESCs after 1 Gy of IR at 2 h, but not at 16 h, post-exposures [31]. Available data suggest that *GADD45A* is implicated in regulation of the G2/M checkpoint after genotoxic stresses [78]; and, this checkpoint was shown to be fully functional in hESC after HD IR exposures [65]. As was the case for *CDKN1A,* induction of *GADD45* and other stress-responsive genes was, in general, much greater after HD IR than after LD IR.

**Figure 6.**
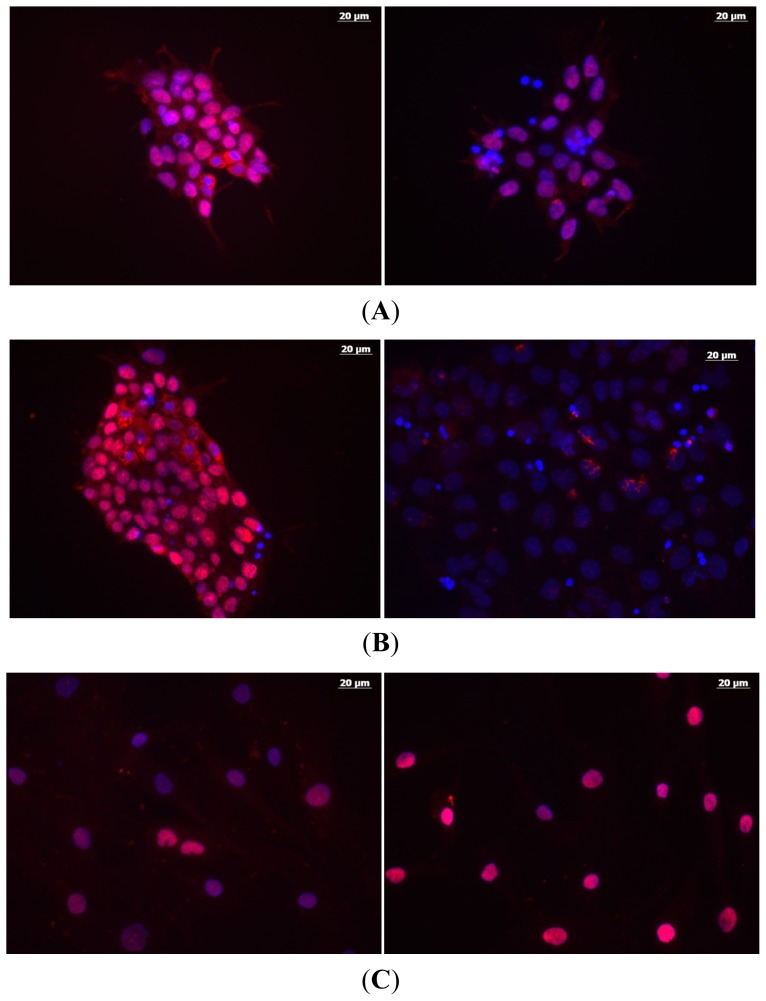
Changes in abundance of p21 protein analyzed in different types of human stem cells after 1 Gy of IR exposures. Shown below are the immunocytochemistry data for cell cultures post treatments (red, p21; blue, nuclei, DAPI staining); left column, sham-IR; right column, 1 Gy. (**A**) H9 hESCs, 4 h post IR; (**B**) H9 hESCs, 24 h post IR; (**C**) hMSCs, 4 h. Bar is 20 μm.

Interestingly, even though the transcript levels of *CDKN1A* were significantly elevated after 1 Gy of IR exposures, we observed a remarkable, almost 2-fold decrease in p21 levels in hESCs cultures as early as 4 h after 1 Gy HD IR (Figure 6A). At 24 h post-exposure, there was about a 3-fold decrease (Figure 6B). In marked contrast, human mesenchymal stem cells (hMSCs) showed a significant increase in p21 after the same treatment (about 2-fold over sham-IR at 4 h, Figure 6C). These findings are in concert with recently published data implicating miRNA-based p21 post-transcriptional regulation [63]. Importantly, the hESC-specific miR-302 family was shown to directly contribute (up to 40%) to a decrease in p21 amount in hESC cultures exposed to genotoxic stresses which, in turn, may prevent the spontaneous differentiation of hESC recovering from such exposures [63]. *CDKN1A* is one of the potent cyclin-dependent kinase inhibitors, and is necessary for robust activation of G1/S checkpoint after IR exposures of different types of human cells *in vitro* [33]. But IR exposures reportedly fail to activate G1/S checkpoint in hESC cultures [65]. Part of the explanation could be that p21 protein levels are reduced after IR exposures in hESCs, as we and others found, perhaps facilitating apoptotic program induction.

It is important to realize that TP53 is implicated in regulation of the majority of IR-responsive genes examined in our study. This key stress-responsive transcription factor was previously shown to be induced *in vivo* by the lowest dose of IR we used in our study (0.01 Gy) following a pattern of increasing activity with the IR dose increase in mouse models [24]. TP53-related mechanisms were shown to be important for LD IR phenomena, such as the adaptive response [79]. It is still not clear why some hESC lines showed statistically significant changes in expression of select stress-responsive genes within the LD IR range, whereas others did not. Part of the explanation could be that the levels and/or activity of transcription factors responsible for regulation of expression of the set of stress-responsive genes we analyzed here, such as TP53, are different in distinct hESC lines. There is some experimental evidence in favor of this assumption [80]. Another possibility might be the inherent differences in susceptibility of distinct hESC lines to apoptosis which seems to be a default pathway on how these cells respond to HDIR. For example, the H1 hESC line is clearly distinct from H7 and H9 hESCs in the manner in which constitutively active Bax is sequestered in the Golgi [81]. But it is not clear how the transcriptional changes in hESCs after LD IR correspond with the cell survival parameters, since hESC death is not readily observed after LD IR. However, previous studies with lymphoblastoid human cells showed that the linear dose-response relationship may not be readily observed within the range of LD IR exposures [82]. This is in concert with what we found for the hESC lines exposed to LD IR in our present work.

The advantages of using a hESC-based model for studying radioresponses, in particular gene expression changes after IR exposure, are numerous. First, pluripotency of hESCs renders them the ability to differentiate into virtually all cell types within human body. Second, hESC can self-renew indefinitely, providing an opportunity for researchers to monitor any delayed effects of IR responses without facing the obstacles of the finite lifespan of normal differentiated human cells. Third, these cells have been shown to be exquisitely sensitive to different types of genotoxic stresses, including HD IR exposures.

## 3. Experimental Section

### 3.1. Cell Cultures and Irradiation

A panel of cultured hESCs (H1, H7, H9 and H14 cell lines, WiCell, Madison, WI, USA) was routinely grown in mTeSR-1 medium (Stemcell Technologies, Vancouver, BC, Canada) on a BD Matrigel hESC-qualified matrix (BD Biosciences, San Jose, CA, USA) at 37 °C and 5% CO_2_. Cell cultures were maintained and propagated according to the supplier’s protocol. Cells were subcultured every 5–7 days using dispase (Stemcell Technologies, Vancouver, BC, Canada). Human bone-marrow derived mesenchymal stem cells (hMSCs, Lonza, Poietics Stem Cells, Walkersville, MD, USA) were used between passages 4 and 5. These cells were grown in Mesenchymal Stem Cell Growth Medium (MSCGM, Lonza, Walkersville, MD, USA) with added l-glutamine (Lonza, Walkersville, MD, USA) and mesenchymal cell growth supplement (Lonza, Walkersville, MD, USA) that was formulated for growing large numbers of hMSCs without inducing differentiation. Cell cultures were grown to 70%–80% confluence, and then subcultured with Trypsin-EDTA (Lonza, Walkersville, MD, USA), per supplier’s protocol as in [83].

The irradiation of cell cultures was performed using an Eldorado 8 ^60^Co teletherapy unit (MDS Nordion, Ottawa, ON, Canada; formerly Atomic Energy of Canada, Ltd). The cells were exposed to 0.01; 0.05; 0.1; and 1 Gy of γ-radiation, and then allowed to recover in a CO_2_ incubator for either 2 or 16 h. In parallel, the control cell cultures were treated with sham-radiation (Eldorado 8 ^60^Co teletherapy unit, MDS Nordion, Ottawa, Ontario, Canada; formerly Atomic Energy of Canada, Ltd.). At the indicated time points post-IR the cell cultures were lysed with TaqMan Gene Expression Cells-to-Ct kit (Life Technologies, Grand Island, NY, USA); and the samples were processed as per supplier’s protocol for downstream analysis.

### 3.2. Immunocytochemistry

The hESC cultures were grown on LabTek Chamber Slide (BD Biosciences, San Jose, CA, USA) as described above. Immunocytochemical analysis was performed essentially as in [29]. Briefly, after treatment with IR, hESCs were fixed with 4% paraformaldehyde for 10 min, and then permeabilized with 0.1% Triton-X-100 for 5 min. Primary monoclonal antibody against human p21 (Clone CP74, Sigma, St. Louis, MO, USA) dissolved in blocking solution containing 3% bovine serum albumin (BSA; Sigma, St. Louis, MO, USA) was added for 1 h. Then fluorescently labeled Alexa Fluor secondary antibodies (Invitrogen, Carlsbad, CA, USA) were used for indirect immunofluorescent detection of p21 protein. All secondary antibodies were previously tested for nonspecific immunoreactivity. DAPI (Invitrogen, Carlsbad, CA, USA) was used to identify the nuclei. The coverslips were mounted with the antifade media (VectaShield, Vector Laboratories, Inc., Burlingame, CA, USA); and the samples were examined by Axioplan Zeiss epifluorescent microscope (Carl Zeiss, Thornwood, NY, USA). The microscope and CCD camera image acquisition settings were continuously kept the same for all corresponding samples.

### 3.3. Quantitative Real-Time PCR and Statistical Analysis

For each gene, qRT-PCR reactions were run three times on one sample. In total, six biological replicates, and three independent technical replicates for each sample were performed for each datapoint, as per TaqMan Gene Expression Cells-to-Ct kit vendor (Life Technologies, Carlsbad, CA, USA). PCR was done on iCycler iQ (Bio-Rad, Inc., Hercules, CA, USA) in 20-μL reactions by using TaqMan Gene Expression assays (Life Technologies, Carlsbad, CA, USA). IDs of the TaqMan Gene Expression assays used are as follows: Hs00355782_m1 (*CDKN1A*), Hs00169255_m1 (*GADD45A*), Hs00427214_g1 (*PCNA*), Hs00198887_m1 (*BTG2*), Hs00248075_m1 (*BBC3*), Hs00902787_m1 (*SESN1*), Hs03044953_m1 (*DDB2*), Hs00275419_s1 (*IER5*), Hs00177725_m1 (*PLK3*), and Hs00171132_m1 (*GDF15*). Each reaction was repeated for 45 cycles; each cycle consisted of denaturing at 95 °C for 15 s, annealing and synthesis at 60 °C for 1 min as per manufacturer’s instructions. Real-time PCR data were analyzed using the comparative CT method within the log-linear phase of the amplification curve obtained for each primers/probe set [14,22,31]. The relative amounts of transcript of all genes analyzed were normalized by 18S rRNA endogenous control primers/probe set, as in previous studies with IR-exposed hESC cultures [30,31]. The average ratios of relative amounts of transcripts in IR-exposed versus sham-treated hESC cultures from three replicate runs were calculated. Data are presented as mean plus/min standard error. Differences were considered statistically significant at *p* value less than 0.05 (Student’s *t*-test).

## 4. Conclusions

In our study, we found experimental evidence that various lines of hESCs, each representing a unique human genome, may respond differently to IR exposures, especially with regard to expression of key stress-responsive genes such as *CDKN1A*, *GDF15* and *BBC3*. In general, the responses of stress genes to LD of IR were much lower than to HD IR given to hESCs. Our data imply no clear linear dose-response relationship within our range of LD of IR. Quite the opposite, our findings may indicate the existence of a threshold for changes in gene expression within LD IR, at least for some human ESC genomes. Even though it is clear that there is no threshold for chemical and molecular damage inflicted by IR exposures because of the physical nature of the phenomenon, the possibility of a threshold in our study may be a reflection of the biological responses of hESC to such low-level IR exposures.
